# Associations between maternal pre-pregnancy BMI and mean diffusivity of the hippocampus and amygdala in infants

**DOI:** 10.1038/s41366-025-01730-8

**Published:** 2025-02-11

**Authors:** Aylin Rosberg, Harri Merisaari, John D. Lewis, Niloofar Hashempour, Minna Lukkarinen, Jerod M. Rasmussen, Noora M. Scheinin, Linnea Karlsson, Hasse Karlsson, Jetro J. Tuulari

**Affiliations:** 1 FinnBrain Birth Cohort Study, Turku Brain and Mind Centre, Department of Clinical Medicine, University of Turku and Turku University Hospital, Turku, Finland; 2 Department of Psychiatry, Turku University Hospital and University of Turku, Turku, Finland; 3 Department of Diagnostic Radiology, Turku University Hospital and University of Turku, Turku, Finland; 4 Program in Neuroscience and Mental Health, SickKids Research Institute, Toronto, ON Canada; 5 Department of Paediatrics and Adolescent Medicine, Turku University Hospital and University of Turku, Turku, Finland; 6 Department of Paediatrics, University of California, Irvine, CA USA; 7 Department of Psychiatry, University of Turku and Satakunta Wellbeing Services County, Turku, Finland; 8 Centre for Population Health Research, Turku University Hospital and University of Turku, Turku, Finland; 9 Department of Clinical Medicine, Unit of Public Health, University of Turku, Turku, Finland; 10 Department of Child Psychiatry, Turku University Hospital, Turku, Finland; 11 Neurocenter, Turku University Hospital, Turku, Finland; 12 Clinical Neurosciences, University of Turku, Turku, Finland

**Keywords:** Neuroscience, Risk factors

## Abstract

**Background:**

Maternal pre-pregnancy obesity may negatively affect offspring outcomes, including neurodevelopment. This study examined the relationship between maternal pre-pregnancy body mass index (MBMI) and the microstructure of the hippocampus and amygdala in neonates.

**Methods:**

Diffusion tensor imaging was used to assess mean diffusivity (MD) in these brain regions in 122 infants (mean gestational age: 39.9 weeks, mean age at scan: 24.8 days) from the FinnBrain Birth Cohort Study (www.finnbrain.fi). Linear regression was applied to explore associations between MBMI and MD at the regional level, while non-parametric permutation analysis was used for voxelwise investigations.

**Results:**

A positive association was found between MBMI and hippocampal MD, particularly in the right hippocampus. Voxelwise analyses showed stronger associations in distinct areas: posterior for the right hippocampus and anterior for the left. No significant association was found between MBMI and amygdala MD.

**Conclusion:**

These findings suggest that in utero exposure to high MBMI may influence hippocampal microstructure in infants, underscoring the need for further research on the intergenerational effects of maternal obesity on early brain development.

## Background

Preclinical and clinical studies link maternal obesity to negative outcomes in the offspring, including cognitive abilities such as learning and memory performance, social behavior [1] and weight profiles [2] in offspring. However, the clinical studies are mostly conducted with children and adolescent populations. To help isolate the effects of prenatal maternal obesity from postnatal factors like diet, lifestyle, and socioeconomic status, recent neuroimaging studies have begun examining how maternal pre-pregnancy body mass index (MBMI) affects infant neurodevelopment [3–5]. We have previously reported on reward structures as a target in infants and showed an association between MBMI and mean diffusivity (MD) of the left caudate nucleus [6], a region involved in hedonic eating, and food-related reward and motivation [7]. MD is a diffusion tensor imaging (DTI) scalar, a specialized magnetic resonance imaging (MRI) technique that estimates the diffusion of water within tissues thereby providing insights into microstructural boundaries to diffusion and underlying cellular structure, and it reflects the average water diffusion. MD is ideal for studying subcortical structures due to the isotropic nature of diffusion in gray matter.

Here we conceptually extend previous analyses to include limbic circuitry based on the importance of limbic circuits in early neurodevelopment and its epidemiological associations with MBMI [8]. The aim is to explore how in utero exposure to high MBMI affects the development of the neonatal limbic system, particularly the hippocampus, a region important in processes such as learning and memory, and the amygdala a region that accounts for the anxiety- and depression-like behaviors associated with MBMI.

## Subjects and methods

### Participants

The DTI data of 122 healthy term-born infants (69 male and 53 female) from the FinnBrain Birth Cohort Study [9] (www.finnbrain.fi) were used in the study. The maternal demographics of pre-pregnancy measures, history of alcohol or drug abuse, severe psychiatric disorders, epilepsy, or related medication use during pregnancy were obtained from the wellbeing services county of Southwest Finland (VARHA) records. The maternal data were based on both self-reported and recorded measurements initially acquired in the maternity clinics as a routine service, although the specific method for obtaining these at the individual level is not specified. None of the mothers had a history of alcohol or drug abuse, severe psychiatric disorders, epilepsy, or related medication use during pregnancy. Demographics of the participants are presented in Table 1. The dataset was the same as our previous study [6], with 6 additional cases.

**Table 1:** Demographic information of mother-infant dyads.

Infant demographics			
	Range	Mean	SD
Birth height (cm)	44–56	50.4	1.86
Birth weight (g)	2530–4700	3485.5	441
Head circumference (cm)	32.5–37.5	35	1.3
Gestational weeks at birth	36.3–42.1	39.87	1.13
Postnatal age at scan (days)	8–45	24.8	7.3
Maternal demographics			
Age at birth (years)	19–41	29.6	4.5
Maternal pre-pregnancy body mass index (BMI)	17.5–38.4	24.3	4
		frequency	
Underweight (BMI < 18)		3	
Normal weight (18 ≤ BMI < 25)		79	
Overweight (25 ≤ BMI < 30)		27	
Obesity (BMI > 30)		13	
Years of formal education			
Missing data		4	
<12 years		34	
12–15 years		36	
15+ years		48	

This study was conducted in accordance with the Declaration of Helsinki, and it was approved by the Ethics Committee of the Hospital District of Southwest Finland (15.03.2011) §95, ETMK: 31/180/2011.

### Image acquisition

MRI data were acquired using a Siemens Magnetom Verio 3T scanner (Siemens Medical Solutions, Erlangen, Germany) with a 12-element Head Matrix coil. Single shell diffusion-weighted data were acquired using a standard twice-refocused Spin Echo-Echo Planar Imaging (SE-EPI) sequence (FOV 208 mm; 64 slices; TR 9300 ms; TE 87 ms). Two mm isotropic spatial resolution was used for the sequence, and the *b*-value was 1000 s/mm. There were in total 96 unique diffusion encoding directions and three b0 images in a three-part multi-scan DTI sequence.

### Imaging analysis

Data quality control was done with DTIPrep [10] tool and visual inspection, and preprocessing steps were completed with various FSL tools. A diffusion tensor model was fitted to each voxel with the DTIFIT tool in FDT (FMRIB’s Diffusion Toolbox) of FSL. The MD values were extracted using the anatomical locations in the FBN-125 template [11]. Detailed information about the imaging data analysis can be found in our previous publications [6, 12].

### Statistical analyses

The associations between MBMI and MD of the hippocampus and amygdala were investigated with two different approaches. A linear regression model that was adjusted for the infant sex and postnatal age in days was used to assess the associations between the MBMI and mean MD of the ROIs (right hippocampus, left hippocampus, right amygdala, and left amygdala). Additional sensitivity analyses were conducted by individually adding the infant birthweight and gestational age at birth in weeks, the mother’s age at birth in years, and socioeconomic status represented by education level (years of formal education) and removing them from the regression model to ensure that the observed associations were not attributable to these variables.

To further investigate the associations and to provide more spatial information about the associations revealed with ROI analysis, a voxel-wise non-parametric permutation analysis was performed with FSL randomise with 5000 permutations and threshold-free cluster enhancement (TFCE) [13]. In a general linear model, the infant sex and postnatal age (in days) were controlled for. Only the voxels reaching the *p* < 0.05 level after TFCE correction were considered significant.

## Results

**Fig. 1:**
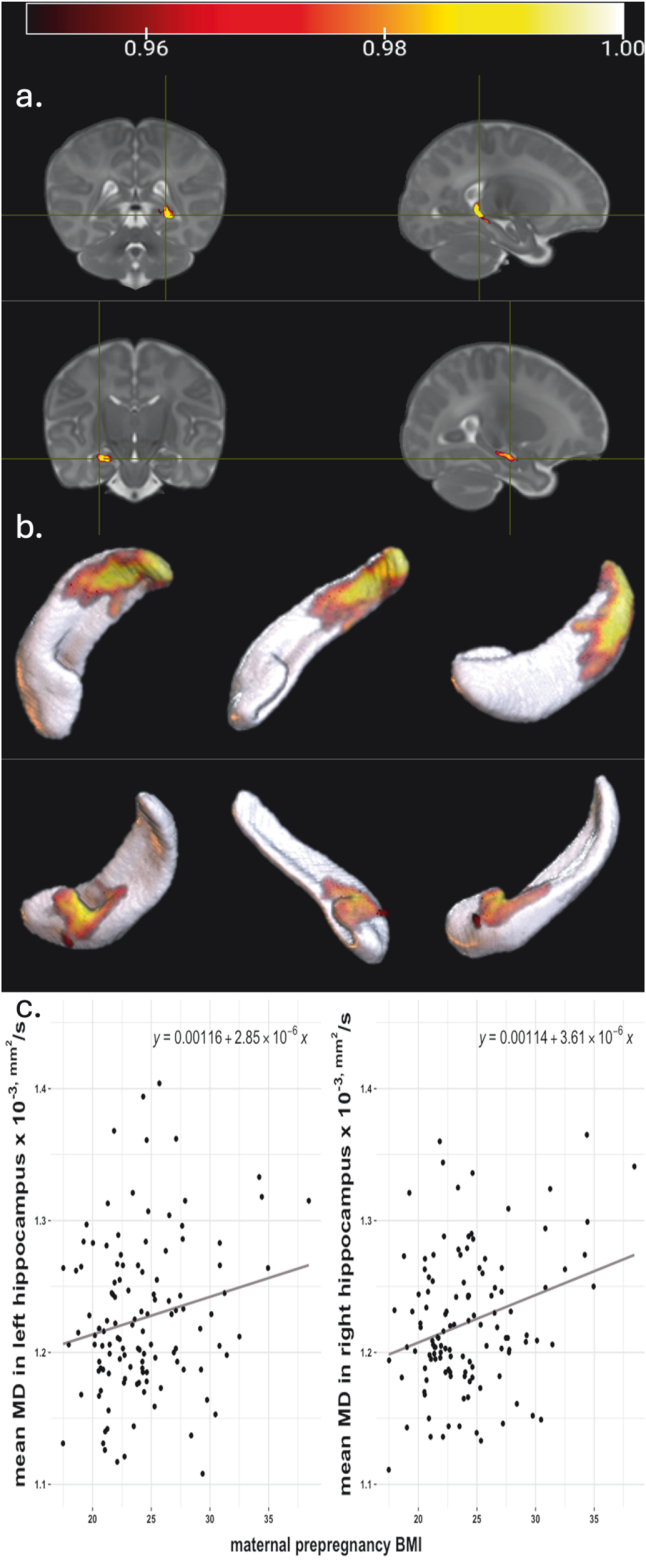
Associations between maternal pre-pregnancy BMI and MD in hippocampi. Positive associations between maternal pre-pregnancy body mass index and infant hippocampus mean diffusivity analyzed with a voxel-wise permutation analysis adjusted for infant sex and age from birth in days presented on **a** a T2 weighted average whole-brain image of the study population and on **b** a 3D rendering of segmented right (up) and left (down) hippocampi. Voxels that are significant (*p* < 0.05) after threshold-free cluster enhancement correction for multiple comparisons are presented in the figure. The color bar represents 1 − *p* values. **a** and b are in neurological convention (right hippocampus top and left hippocampus bottom). Same associations presented with a scatterplot (**c**) for left hippocampus (left) and right hippocampus (right).

A positive association was observed between MBMI and the mean MD in the right hippocampus (*beta* = 4.084 ± 1.341 (×10^−6^), *t* = 3.045, *p* = 0.00287) (see Fig. 1). The positive association between MBMI and the left hippocampus mean MD (*beta* = 3.670 ± 1.475 (×10^−6^), *t* = 2.489, *p* = 0.0142) did not survive the FDR correction. There were no associations between MBMI and amygdala MD (right: *beta* = 1.190 ± 1.037 (×10^−6^), *t* = 1.147, *p* = 0.254; left: *beta* = 6.683 ± 4.795 (×10^−7^), *t* = −0.868, *p* = 0.387).

Voxelwise permutation tests, adjusted for infant’s sex and age from birth in days, yielded significant voxels in the posterior right hippocampus and anterior left hippocampus (see Fig. 1). No significant voxels were detected in the amygdalae. None of the tests yielded sex differences.

## Discussion

Our study revealed a positive association between MBMI and MD in the posterior right hippocampus and anterior left hippocampus. MD represents the average movement of water molecules within a voxel and reflects how freely water can diffuse in different directions. Since cell walls act as a barrier to diffusion higher tissue density results in lower MD values. As the brain matures, it is expected that MD will decrease due to the growing presence of restrictive elements such as increased axon diameter and increased dendritic structure [14]. Hence, the increase in MD values as the MBMI gets higher might suggest a delay in infant neurodevelopment.

The findings indicate associations between MBMI and MD in different locations in right hippocampus and the left hippocampus. This lateralization aligns with both clinical and preclinical evidence of normal hippocampal functional asymmetry, which begins in embryonic development and persists throughout lifespan [15]. Therefore, the lateralization observed in the findings might simply be due to normal functional lateralization of the hippocampus.

The amygdala is one of the earliest developing structures in the human brain and known to be selectively sensitive to exposures of prenatal stress [16–19] including the stress of gestational malnutrition [20]. However, we found no statistically significant associations between MBMI and amygdala MD. The null finding suggests region-specific impact on infant neurodevelopment. The small size of the amygdala and the resolution (2 mm isotropic) used in the study might have contributed to the null finding. More research is needed to reveal the selective effect mechanisms between in utero exposure to high MBMI and neurodevelopment.

It is worth noting that we had the data on maternal gestational diabetes mellitus (GDM) and whether they had started using insulin during the index pregnancy. Due to the small number of GDM cases (*n* = 18), and mothers who started using insulin (*n* = 1) and our previous research showing that GDM did not impact the results [6] we included the mothers with GDM.

There are some limitations. The study data were collected exclusively from the Finnish population. It is essential to replicate similar studies involving participants with more diverse backgrounds. The data on paternal BMI, maternal gestational weight gain and diabetes prior to pregnancy could create a more comprehensive understanding on the subject.

## Conclusions

Our results revealed a positive association between MBMI and MD in the infant bilateral hippocampi. Despite the limitations of our study, these findings highlight the importance of intrauterine environment, including maternal weight profile, on offspring neurodevelopment. Assessing the long-term outcomes of in utero exposure to high MBMI and its interaction with different lifestyle factors are left for future studies.

## Data Availability

The current Finnish legislation and our Ethical Board approval do not permit the open data sharing of the imaging data or derived measures. Investigators interested in getting access to the data are encouraged to contact FinnBrain’s Principal Investigators (https://sites.utu.fi/finnbrain/en/contact/). The analysis code can be made available upon a reasonable request to the corresponding author.
